# Muscle strength differences in healthy young adults with and without generalized joint hypermobility: a cross-sectional study

**DOI:** 10.1186/s13102-016-0037-x

**Published:** 2016-04-25

**Authors:** Pranay Jindal, Amitesh Narayan, Sailakshami Ganesan, Joy C. MacDermid

**Affiliations:** McMaster University, Hamilton, ON Canada; Kasturba Medical College, Mangalore, Karnataka India

**Keywords:** Generalized joint hypermobility (GJH), Isometric Muscle Strength, BTe RS primus isokinetic dynamometer, Beighton and Horan Joint Mobility Index, India

## Abstract

**Background:**

Generalized joint hypermobility (GJH), in the absence of symptoms, is a common clinical finding. The joint instability present due to excessive musculoskeletal flexibility in hypermobile joints impairs the external force production during muscle contraction. However, whether GJH is associated with muscle weakness is unclear. This study evaluated differences in upper and lower limb muscle strengths among asymptomatic young adults with and without GJH.

**Methods:**

One hundred six young adults (53 hypermobile, i.e. 25 male (mean age 22 ± 1.8); 28 female (mean age 21 ± 1.8), and 53 non-hypermobile, i.e. 25 male (mean age 19 ± 1.06); 28 female (mean age 20 ± 1.4) were selected using a cut-off ≥ 4 on Beighton and Horan Joint Mobility Index. Isometric strength of elbow and knee extensors was measured using an isokinetic dynamometer. Independent sample t- tests were done to compare the muscle strengths of hypermobile and non-hypermobile participants. One-way ANCOVA was applied to control the effect of height and body mass on muscle strength.

**Results:**

Male hypermobile participants had significantly less strength than non-hypermobile males in the right (71.7 Nm, SD = 23.1, vs 97.6 Nm, SD = 47.4, *p* = 0.006*) and left (74.8 Nm, SD = 24.3, vs 97.7 Nm, SD = 45.5, *p* = 0.007*) elbow extensors and right knee extensors (188.7 Nm, SD = 83.3, vs 228.3 Nm, SD = 106.7, *p* = 0.03*). In females, both elbow extensors (right: 51.9 Nm, SD = 16.2 vs 48.8 Nm, SD = 17.8, *p* = 0.4; left: 48.9 Nm, SD = 17.2, vs 44.7 Nm, SD = 15.1, *p* = 0.2) and knee extensors (right: 161.3 Nm, SD = 74.9 vs 145.5 Nm, SD = 75.8, *p* = 0.3; left: 155.2 Nm, SD = 73 vs 124.3 Nm, SD = 69.6, *p* = 0.07) strength were not statistically different between hypermobile and non-hypermobile participants.

**Conclusion:**

The findings indicate that male participants with GJH have less isometric muscle strength in both elbow extensors and right knee extensors compared to non-hypermobile male participants. Female hypermobile participants did not show any significant differences in muscle strength compared to non-hypermobile female participants.

**Electronic supplementary material:**

The online version of this article (doi:10.1186/s13102-016-0037-x) contains supplementary material, which is available to authorized users.

## Background

Generalized joint hypermobility (GJH) is defined as “a condition in which most of the individual’s synovial joints move beyond the normal limits of the range of motion, taking into consideration the age, gender and ethnic background in otherwise healthy subjects” [1]. The genetic make-up of an individual determines the extent of GJH [2]. Ligamentous extensibility is a result of changes in the connective tissue like collagen, elastin, fibrillin, and tenascin [1, 2]. In females, hormones like relaxin also affect the ligament extensibility [3].

Epidemiologically 10 % of the Caucasian and 25 % of the African and Asian population has GJH [4–6]. Females are three times more likely to be hypermobile compared to males at any age [7–12], and hypermobility decreases with increasing age due to tissue stiffening [9, 11]. The reduction in joint hypermobility is more rapid during childhood, lesser in teenage years and very slow during adult life [13–15]. The non-dominant side (usually left) is typically more hypermobile than the dominant side (usually right) [7, 9].

Despite debate about the acceptability of the test maneuvers and components [16, 17], the Beighton and Horan Joint Mobility Index (BHJMI) is the most commonly used tool to measure GJH and has good to excellent reliability [16, 18]. The BHJMI defines hypermobility based on how many of the joints used in the index have a range of motion indicating hypermobility, resulting in a score between 0 and 9 [9, 18]. There are no universally accepted cut-off scores for diagnosing GJH [16, 17]; however, many define GJH as present if BHJMI score is ≥ 4 of 9 [7, 8, 19–24].

Many individuals with GJH have no symptoms or disability [25], and it may even serve as an advantage for professionals like ballet dancers [26] and musicians [27]. However, approximately 3.3 % women and 0.6 % men with GJH tend to develop pain in lower limbs and shoulder joints [10, 20, 25, 28]. Recent studies also suggest that young females with GJH have lower levels of physical fitness [29] and have decreased walking and jumping capacity [23]. Prospective cohort studies in pre-adolescent school-age children have shown that it takes 4 years for GJH to become symptomatic (painful) in lower limbs and shoulder joints [30, 31]. Studies also suggest that GJH in children can be a risk factor for developing joint pain later in adolescent years [32]. The symptomatic form of GJH is called benign joint hypermobility syndrome (BJHS). BJHS is characterized by generalized joint laxity with associated musculoskeletal complaints (arthralgia, recurrent subluxations or dislocations after an acute trauma, childbirth or change in physical activity) in the absence of any systemic, rheumatic, neurological, skeletal or metabolic disease [33]. Individuals with BJHS are found to have decreased physical fitness [34], poor joint proprioception [35] and muscle weakness [36]. Since BJHS is sequelae of GJH, it is possible that individual with GJH may also have muscle weakness and that this may place them at risk of future injury or disability.

There are limited studies and conflicting evidence on muscle strength in young asymptomatic hypermobile individuals [22, 24, 36–39]. All existing studies included only Caucasians [22, 24, 36–39], and most researchers [24, 36–39] have studied the pooled (right + left) muscle strength in the lower limb. Lower limb joints have more bony stability than upper limb joints, and there is less reliance on soft tissues for stability. Thus, effects of hypermobility may be more pronounced in upper limbs compared to lower limbs. Also, upper limbs are more mobile compared to lower limbs, and the impact of hypermobility coupled with decreased muscle strength could lead to work-related upper extremity disorders. Thus, the effects of hypermobility may differ between upper and lower limbs. The Dominant side is generally stronger [36, 40–43] and less hypermobile [7, 9], thus pooling the muscle strength of dominant and non-dominant side might have diminished the existing strength differences in dominant and nondominant extremities.

Asian and African populations with GJH have greater ranges of joint mobility as compared to Caucasians [9, 10, 44], and thus, might be more prone to injury and complications arising from GJH. However, there is a lack of literature assessing the effect of GJH on asymptomatic Asian young adults. Thus, an information gap exists whether there are muscle strength differences between hypermobile and non-hypermobile young Asian individuals in both the upper and lower limbs. Therefore, this study evaluated strength differences between individuals who exhibit GJH and those who do not in two key muscle groups (elbow and knee extensors) among age and gender matched healthy young adults, and controlling for the effect of height, body mass and limb dominance.

## Methods

Institutional ethics committee (No. IEC/KMC/09/2007-2008) at KMC Mangalore, India, approved the study. All participants were recruited from a graduate student hostel in the southern part of India. After obtaining the written informed consent, 200 healthy volunteer participants aged 18–25 years were screened for GJH using BHJMI (Additional file 1). Using convenience sampling, we identified 53 hypermobile participants. We also recruited 53 non-hypermobile subjects of the same age. Exclusion criteria included: Involvement in more than 6 h per week of physical activities, any diagnosis of autoimmune disorders, connective tissue disorders, recent traumatic injury, musculoskeletal disorders, neurological disorders, as well as pain experienced currently or during the past 2 weeks.

The dominant upper limb was determined by asking participants about the preferred hand while writing. The dominant lower limb of the participants was determined by asking participants about the preferred lower limb while kicking a soccer ball [45]. In this study, a cut-off score of ≥ 4 on BHJMI was considered as hypermobile (for both males and females). A physiotherapist administered the protocol suggested by Juul-Kristensen [46] (Appendix 1) for screening. Both hypermobile and non-hypermobile participants were assessed for isometric muscle strength for right and left elbow extensors and knee extensors on Baltimore Therapeutic Equipment (BTe), Primus RS isokinetic dynamometer. The BTe RS primus isokinetic dynamometer was calibrated weekly during the testing. The same physiotherapist performed the hypermobility screening, testing and the strength evaluation in an out-patient setting.

A practice trial was provided to the participants to get accustomed to the testing maneuver. No verbal prompts were given during the strength testing. Participants were advised to stop the test immediately if they experienced any unusual pain or discomfort. As our participants were young and healthy, a 5-second rest was given between each trial to avoid fatigue. A 2 min rest was given after testing a muscle group or side, before testing the next muscle group. Ten minutes of rest was given after completing the testing procedure and participants were enquired for any pain or discomfort. The following sequence was followed for muscle strength testing: right elbow extensors, left elbow extensors, right knee extensors and left knee extensors. Standardized testing procedures and tool/pad number 701, as per the BTe RS Primus instruction manual, were used.

### Test positions and procedure for Isometric muscle strength testing:

1. Test positions and procedure for elbow extension: Participants were asked to stand in front of the exercise head of the dynamometer with their feet shoulder width apart. Shoulders were in neutral rotation, flexion, and abduction. The Dynamometer axis was aligned with the lateral epicondyle of the elbow joint. The elbow was positioned in 90° flexion, with the forearm supinated, the wrist in a neutral position and the hand was placed centrally on the handle of the tool (Appendix 2, image 1). Participants were instructed to extend their elbow (exert pressure downwards on the pad) to exert a maximum contraction on the “Go” command of the examiner for 3 s.
2. Test position and procedure for knee extension: Participants were tested in a sitting position on a chair with a backrest. The anatomical axis of rotation of the knee joint was aligned with the dynamometer axis, and the pad of the tool was positioned centrally at the lower part of the shin of the tibia. The knee was kept at 90° flexion, the hip in neutral rotation and abduction, and the foot was positioned in plantar flexion. The hands were placed on the abdomen and the trunk, hips, and mid-thigh were stabilized on the chair by Velcro straps (Appendix 2, image 2). Participants were instructed to extend their knee (exert pressure upwards on the pad) and to exert a maximum contraction on the “Go” command of the examiner for 3 s.

Three criterion trials of maximum voluntary isometric contraction (MVIC) were made for elbow and knee extensor, and peak torque measurements were recorded in newton-meters (Nm). The largest value of three trials represented the participants’ peak torque.

### Statistical analysis

Data were entered and checked for quality by random re-checking of the original data against the electronic data file. Data were analyzed using SPSS v (21) software with *p* value set at 0.05. All statistical tests were two-tailed, and the statistical power was calculated post-hoc. Data were examined and accepted as being sufficiently normal if the skewness and kurtosis were within ±2 SD. The descriptive statistical analysis was done using independent samples t-test to obtain mean age (years), body mass (kg.), standing height (cms.), and Beighton scores for hypermobile and non-hypermobile participants. Gender and body size have an influence on muscle strength [47–49]; hence, the data were analyzed in two ways to help delineate factors associated with differences. First, the data for hypermobile and non-hypermobile participants were separated by gender, and independent sample t-tests were used to compare the subgroups for elbow and knee extensor strength. In the second analyses, one-way analysis of covariance (ANCOVA) was performed comparing hypermobile versus non-hypermobile strength scores across all participants, but controlling for the height and body mass (covariates). Analyzes were conducted separately for right and left elbow extensors and right and left knee extensors.

## Results

One hundred six participants completed the testing without difficulty. All of the participants except one were right upper and lower limb dominant. None of the participants reported ambidexterity, any pain or discomfort during or after the strength testing. Table 1 shows the descriptive statistics of body mass, height, and Beighton scores of both groups.

**Table 1:** Description of study participants

Variables	Males (*n* = 50)		*p*	Females (*n* = 53)		*p*
	Hypermobile (*n* = 25)	Non-hypermobile (*n* = 25)		Hypermobile (*n* = 28)	Non-hypermobile (*n* = 28)	
Mean age (years)	22 ± 1.8	19 ± 1.06	0.01	21 ± 1.8	20 ± 1.4	0.7
Mean body mass (kg)	67.7 ± 11.4	61.4 ± 9.1	0.03	55.4 ± 6.4	55.2 ± 8.8	0.9
Mean standing height (cm)	170.4 ± 6.1	163.2 ± 22.2	0.1	160.4 ± 7.3	158.0 ± 5.9	0.1
Median BHJMI scores	5	0	NA	5	2	NA

Independent sample t-tests showed that hypermobile males were significantly weaker compared to non-hypermobile males with respect to elbow extensor strength for both right (*p* = 0.02) and left (*p* = 0.03) extremities (Table 2, unadjusted mean strength). After controlling for height and body mass in ANCOVA analysis, there were large and statistically significant differences between male hypermobile and non-hypermobile participants for both right (*p* = 0.006) and left (*p* = 0.007) elbow extensors and the right knee extensors (*p* = 0.03) (Table 2, adjusted mean strength). Female participants did not have significant differences based on hypermobility, even after controlling for height and body mass. In males, height and body mass (covariates) accounted for 10 % of the variance in elbow strength and 9 % for right knee extensor strength (Table 2).

**Table 2:** Comparison of isometric strength of elbow and knee extensors between Hypermobile (H) and Non-Hypermobile (NH) participants after one-way ANCOVA (height and body mass were selected as covariates)

Muscle group	Gender	Category	N	Unadjusted mean strength (Nm) ± SD (without controlling for covariates)	*p* value	Adjusted mean strength (Nm) (after controlling for covariates)	95 % CI		F	Partial eta squared
							Lower	Upper		
Elbow extensors right	Female	H	28	51.9 ± 16.2	*p* = 0.4	51.1	45	57.2	*p* = 0.7	0.002
		NH	28	48.8 ± 17.8		49.6	43.5	55.6		
	Male	H	25	71.7 ± 23.1	*P* = 0.02*	69	53.8	84.2	*p* = 0.006*	0.1
		NH	25	97.6 ± 47.4		100.3	85.1	115.5		
Elbow extensors left	Female	H	28	48.9 ± 17.2	*p* = 0.26	48	42.4	53.7	*p* = 0.5	0.007
		NH	28	44.7 ± 15.1		45.5	39.9	51.2		
	Male	H	25	74.8 ± 24.3	*p* = 0.03*	71.5	56.9	86.1	*p* = 0.007*	0.1
		NH	25	97.7 ± 45.5		101	86.4	115.7		
Knee extensors right	Female	H	28	161.3 ± 74.9	*p* = 0.37	158.2	130.8	185.5	*p* = 0.6	0.005
		NH	28	145.5 ± 75.8		148.6	121.2	175.9		
	Male	H	25	188.7 ± 83.3	*p* = 0.13	178.8	141	216.6	*p* = 0.03*	0.09
		NH	25	228.3 ± 106.7		238	200.3	276		
Knee extensors left	Female	H	28	155.2 ± 73	*p* = 0.07	152.3	126.3	178.4	*p* = 0.1	0.03
		NH	28	124.3 ± 69.6		127.1	101.1	153.1		
	Male	H	25	178.1 ± 96.2	*p* = 0.23	169.9	130.1	209.6	*p* = 0.08	0.06
		NH	25	212.9 ± 101.2		221.1	181.1	260.9		

* *p* < 0.05 denotes significant findings

## Discussion

In this study, among males, hypermobile participants demonstrated less isometric strength for right and left elbow extensors and right knee extensors than non-hypermobile participants. The differences were even more statistically significant after removing the influence of height and body mass as covariates. Controlling for these covariates increases our confidence that hypermobility underpinned the decreased elbow and knee extensor muscle strength. Amongst females, there were no statistically significant differences between hypermobile and non-hypermobile participants in elbow and knee extensor strength. Further, since this effect was retained after controlling for height and body mass, we are confident that there was no evidence of hypermobility compromising female extensor strength in this study.

The present study adds to the current knowledge as it establishes that males have weaker elbow extensors strength in the presence of hypermobility. Scheper et al. [22] found decreased grip and shoulder abductor strength in healthy young adults with GJH. Since there is a higher risk of sports injuries in the upper limb in individuals with GJH [50], muscle weakness could be a contributing factor for increased risk of upper limb injuries during sports.

In this study, after controlling for body size, hypermobile males demonstrated less isometric strength of the right knee extensors compared to non-hypermobile males. Isometric strength of left knee extensors among hypermobile and non- hypermobile males, although it did not reach statistical significance (*p* = 0.08), the trend was in a similar direction as of right knee extensors. Our findings support a previous study showing that knee extensors are weak in hypermobile young males [22]. Our results are in contrast to studies by Jensen et al. [24], Stewart and Burden [37] and Kristensen et al. [38] who found no difference in knee extensor strength among hypermobile and non-hypermobile individuals. Stewart and Burden [37] included athlete males where sports participation might have influenced the muscle strength. Our sample was not actively involved in sports activities. Thus, it was easier to detect muscle strength differences that were not mitigated by sports specific training. Studies by Jensen et al. [24] and Kristensen et al. [38] included adult participants with knee joint pain, and Jensen et al. [24] found no differences in knee extensor strength in individuals with hypermobility. However, Kristensen et al. [38] found decreased knee extensor strength in adult females. Since males have high pain tolerance [51, 52], it could be possible that pain did not affect the muscle strength in males as compared to females who have less pain tolerance. Since our sample was young and asymptomatic, it could be possible that muscle strength differences are related to joint pain in hypermobile individuals. Studies by Jensen et al. [24] and Kristensen et al. [38] included adult males with mean age of 40.1 to 40.3 years. The extent of hypermobility decreases as age increases [9, 11], it could be possible that our sample of young adults (mean age 21 years) was more hypermobile and weaker compared to the adult population in Kristensen’s and Jensen’s study.

Jensen et al. [24] and Kristensen et al. [38] reported the pooled muscle strength in lower limbs, which might have influenced the strength differences in right and left extremities. Importantly, Kristensen et al. [38] compared the strength of hypermobile knee extensors (right or left) to dominant lower limb knee extensor strength in non-hypermobile adults. The effects of pain and dominance may have contributed to the variation that would have made it harder to detect the difference in muscle strength in earlier studies. Studies suggest that the dominant side has greater strength than the non-dominant side, [36, 40–43] and the dominant side has also been reported to be less hypermobile compared to the non-dominant side [7, 9]. Although our participants were healthy and young, decreased knee extensor strength at a young age could be of clinical relevance as they may be more prone to injuries. Recent studies show an increased risk of the knee joint injuries during contact sports in participants with GJH [53, 54].

In our study amongst females, there were no statistically significant differences between hypermobile and non-hypermobile participants with respect to right or left elbow and knee extensor strength. Our results are similar to studies by Jensen et al. [24], and Mebes et al. who found no difference in knee extensor strength among hypermobile and non-hypermobile females. Scheper et al. [22] reported decreased knee extension strength (pooled) in asymptomatic young females. Mebes et al. suggested that muscles in hypermobile women have a higher rate of force development as compared to non-hypermobile women. This higher rate of force development may counteract a lack of stability in hypermobile joints. Force development is an important factor for joint stabilization, and since individuals with GJH have less joint stability due to lax passive structures [55] they might be relying on neuromuscular mechanisms such as force development for more joint stability. We did not measure the rate of force development in our study, so we could not determine if this was used to counteract the impact of hypermobility on knee extensor strength in our sample. Our participants reported participated in less than 6 h of physical activity; however, we did not collect data on specific type and duration of physical activity participation. It could be possible that the type and duration of physical activity influenced the muscle strength among male and female participants.

Methodological and sampling issues in previous studies comparing the dominant side to the non-dominant side and pooling muscle strength values may have reduced the potential to find statistically significant differences in muscle strength among hypermobile and non-hypermobile participants compared to our study in which we have a younger sample that was controlled for gender and anthropometrics. Evidence suggests that with increasing age there is a decline in muscle strength [43, 56], and hypermobility [9, 11]. Given these trends, the impact of hypermobility may be more pronounced in younger individuals. We compared the right (dominant) side of hypermobile participants to the right side of non-hypermobile participants (dominant) and did the same for the left side which increased the likelihood of finding statistically significant differences.

A moderate statistical power due to small sample size in the present study (*N* = 106, 25–28 in each group) may have played a role in limiting the significance of some of the statistical comparisons conducted. On the post-hoc power analysis on the basis of between-groups means, and standard deviation observed in the present study, a sample size of approximately 50–70 participants per group would be needed to calculate 20 % difference in muscle strength (assuming 20 % difference in strength is clinically important).

There is sufficient evidence that hypermobility, particularly at the elbow, is associated with strength deficits in males. Reduced joint stability in combination with reduced muscle strength could be a major etiological factor in the development of upper extremity work-related disorders or sports injury and should be investigated further for prevention and management. Higher risk of upper limb [50] and lower limb sports injuries [53, 54, 57–59], pain [60] and decreased dynamic trunk stability [61] have been reported in individuals with GJH. These studies in combination with the findings of the present study suggest that a more detailed routine examination of GJH is needed during the investigation of musculoskeletal disorders.

### Limitations

Our study findings should be considered in light of some inherent limitations. As our sample included only young participants, our results cannot be generalized to older adults. Secondly, we assessed only two different joints and the associated muscle strength thus we cannot generalize these findings to all upper extremity and lower extremity joints. Finally, the trends in the lower limb strength of males were consistent with the direction of the significant findings for the upper limb strength but were statistically significant only on one lower limb. It is possible that with a larger sample size these differences would have consistently reached statistical significance. We used a smaller rest period of 5 s between each strength testing trials for knee and elbow extensors, which might have influenced the muscle strength in our sample. Some statisticians suggest Bonferroni corrections to adjust for multiple *p* values; however, it is debatable [62]. Given our small sample size, we felt that to do a Bonferroni correction would unnecessarily increase type 2 errors. Our analytical approach did not use an overall omnibus tested which would have reduced the probability of Type 1 error, but rather performed sex-disaggregated and ANCOVA analyses which we used to provide additional insights into the nature of the differences between males and females with respect to hypermobility in different muscle groups.

### Implications for future research

Large cross-sectional studies are needed to explore the effect of GJH in other muscle groups. These should include testing of multiple joints of the upper and lower limbs and should consider a broad age range, activity levels, and include dynamic strength testing in both genders. The effects of gender and limb dominance should be analyzed separately since differential effects may occur. Studies might also explore the relationship between severity of GJH and muscle strength, and could further investigate if there are differences between Caucasians and Asian strength taking into account their level of hypermobility. Future studies can also explore the relationships between participants’ level and type of physical activity, hypermobility and muscle strength.

## Conclusion

From the present study it can be concluded that Asian males with GJH have less isometric strength in both elbows and right knee extensors compared to non-hypermobile males. The impact of GJH in Asian females is not the same as for men and hypermobility appears to be a less important factor influencing muscular strength.

### Consent to publish

Consent to publish the results was obtained from the participants.

### Availability of data and materials

The dataset supporting the conclusions of this article is included within the article as an additional file (Additional file 2).

## Figures and Tables

**Fig. 1:**
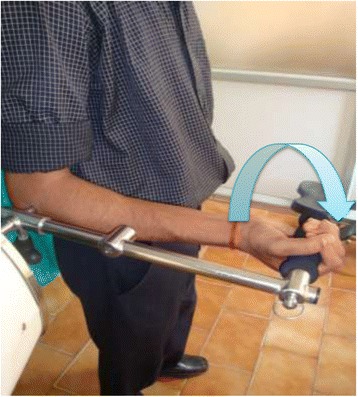
Testing position for elbow extensors

**Fig. 2:**
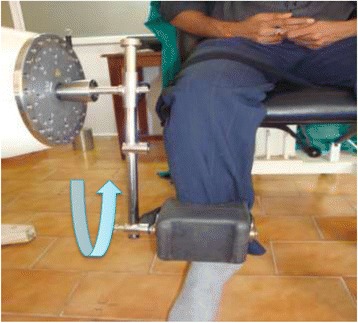
Testing position for knee extensors

## Appendix 1

### Test protocol for Beighton and Horan Joint Mobility Index testing

1. Passive apposition of the thumb to the flexor side of the forearm (shoulder 90° flexed, elbow extended and hand pronated), tested on right and left side, is performed by the patient after the following procedure. The examiner performs the test and asks: ‘Can you with a straight arm move your thumb down so it touches the lower part of the forearm?’ If the test is negative, meaning no touch, the examiner asks: ‘Have you been able to do this previously?’
2. Passive dorsiflexion of the little finger > 90° (elbow flexed 90°, the forearm and hand pronated resting on a table), tested on the right and left side, is performed by the patient after the following procedure. The examiner performs the test and asks: ‘Can you with the forearm resting on the table, move your little finger, so it is pointing a little bit backwards?’ If the test is negative, the examiner asks: ‘Have you been able to do this previously?’
3. Passive hyperextension of the elbow >10° (shoulder 90° abducted and hand supinated), tested on the right and left side, is performed by the patient after the following procedure. The examiner performs the test and asks: ‘How much are you able to overstretch your elbow in this position (illustrated by the examiner) with your palm pointing towards the roof?’ If the test is negative, meaning no overstretching, the examiner asks: ‘Have you been able to overstretch the elbow previously?’
4. Passive hyperextension of the knee >10° (standing), tested on right and left side, is performed by the patient after the following procedure. The examiner performs the test and asks: ‘How much are you able to overstretch your knee when you are standing straight up?’
  If the test is negative, meaning no overstretching, the examiner asks: ‘Have you been able to overstretch the knee previously?’
5. Forward flexion of the trunk, with knees straight, so that the palms of the hands rest easily on the floor, is performed by the patient after the following procedure. The examiner performs the test and asks: ‘Can you with straight knees bend your body forward and place both palms easily on the ground?’ If the test is negative, meaning no touch on the ground with the whole palm of the hands, the examiner asks: ‘Have you been able to do this previously?’

## Additional files

Additional file 1: Beighton and Horan Joint Mobility Index. (DOCX 24 kb) Additional file 2: Additional data file number 2. (XLS 39 kb)
