# Supplementary material for: Muscle strength differences in healthy young adults with and without generalized joint hypermobility: a cross-sectional study
Source: BMC Sports Sci Med Rehabil. 2016 Apr 25;8:12. doi: 10.1186/s13102-016-0037-x (PMC4845357; doi:10.1186/s13102-016-0037-x)
Supplement: Additional file 1: — Beighton and Horan Joint Mobility Index. (DOCX 24 kb) [file 13102_2016_37_MOESM1_ESM.docx]

**Appendix 1- Beighton and Horan Joint Mobility Index**

1.  More than 10º hyperextension of the elbow.

R L
2.   Passively touch the forearm with the thumb, while flexing the wrist.
 R L

3. Passive extension of the fingers or a 90º or more extension of the fifth finger.

R L

4.   Knees hyperextension greater than or equal to 10º.

R L

5. Touching the floor with the palm of the hand when reaching down without bending the knees.

**Scoring:** One point is given for each of the five simple tests. Tests are done on the arm and lower limb on both sides of the body, It is scored between 0 and 9; 1 point is given if the criterion is met and 0 if it is not. A score of 4 or more suggests hypermobility.
